# Aminoglycoside binding and catalysis specificity of aminoglycoside 2″-phosphotransferase IVa: A thermodynamic, structural and kinetic study

**DOI:** 10.1016/j.bbagen.2016.01.016

**Published:** 2016-04

**Authors:** Elise Kaplan, Jean-François Guichou, Laurent Chaloin, Simone Kunzelmann, Nadia Leban, Engin H. Serpersu, Corinne Lionne

**Affiliations:** a CNRS, FRE3689 — Université de Montpellier, Centre d'études d'agents Pathogènes et Biotechnologies pour la Santé, F-34293 Montpellier, France; b CNRS, UMR5048 — Université de Montpellier, Centre de Biochimie Structurale, F-34090 Montpellier, France; c INSERM, U1054, F-34090 Montpellier, France; d Francis Crick Institute, Mill Hill Laboratory, London NW7 1AA, UK; e Department of Biochemistry, Cellular and Molecular Biology, University of Tennessee, Knoxville, TN 37996, USA

**Keywords:** APH(2″), aminoglycoside 2″-phosphotransferases, APH(3′), aminoglycoside 3′,5″-phosphotransferases, *LE*, ligand efficiency, N_CNO_, number of non-hydrogen atoms of the ligand, PMF, potential of mean force, Aminoglycoside modifying enzymes, Transient kinetics, Isothermal titration calorimetry, Electrostatic potential calculations, Molecular dynamics simulations, X-ray crystallography

## Abstract

**Background:**

Aminoglycoside *O*-phosphotransferases make up a large class of bacterial enzymes that is widely distributed among pathogens and confer a high resistance to several clinically used aminoglycoside antibiotics. Aminoglycoside 2″-phosphotransferase IVa, APH(2″)-IVa, is an important member of this class, but there is little information on the thermodynamics of aminoglycoside binding and on the nature of its rate-limiting step.

**Methods:**

We used isothermal titration calorimetry, electrostatic potential calculations, molecular dynamics simulations and X-ray crystallography to study the interactions between the enzyme and different aminoglycosides. We determined the rate-limiting step of the reaction by the means of transient kinetic measurements.

**Results:**

For the first time, *K*_d_ values were determined directly for APH(2″)-IVa and different aminoglycosides. The affinity of the enzyme seems to anti-correlate with the molecular weight of the ligand, suggesting a limited degree of freedom in the binding site. The main interactions are electrostatic bonds between the positively charged amino groups of aminoglycosides and Glu or Asp residues of APH. In spite of the significantly different ratio *K*_d_/*K*_m_, there is no large difference in the transient kinetics obtained with the different aminoglycosides. We show that a product release step is rate-limiting for the overall reaction.

**Conclusions:**

APH(2″)-IVa has a higher affinity for aminoglycosides carrying an amino group in 2′ and 6′, but tighter bindings do not correlate with higher catalytic efficiencies. As with APH(3′)-IIIa, an intermediate containing product is preponderant during the steady state.

**General significance:**

This intermediate may constitute a good target for future drug design.

## Highlights

• Aminoglycosides with amino groups in 2′ and 6′ are best ligands for APH(2″)-IVa.
• Binding of aminoglycosides on APH is mainly driven by electrostatic interactions.
• Enzyme affinity seems to anti-correlate with the molecular weight of the ligand.
• With different aminoglycosides, the ratio *K*_d_/*K*_m_ varies, but not transient kinetics.
• A product release step is rate-limiting for the overall reaction.

## Graphical abstract

**Figure:**
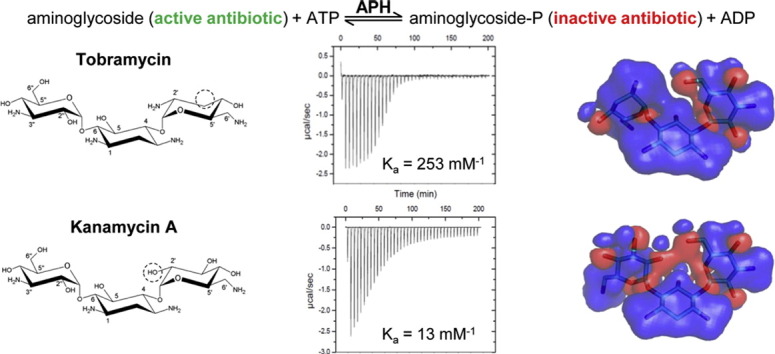

## 1 Introduction

Bacterial infections are the cause of diseases that are often fatal and highly contagious. The generalized introduction of antibiotics after the Second World War was one of the most important therapeutic progresses of the 20th century. However, the misuse and over use of antibiotics in the past decades have led to the emergence of several resistant bacteria strains [1]. Bacteria have developed several strategies to combat antibiotics. Of these, the mechanisms whereby bacteria express enzymes that chemically inactivate antibiotics such as aminoglycosides are of particular interest. There are more than 120 bacterial enzymes that inactivate aminoglycosides by *N*-acetylation, *O*-adenylation or *O*-phosphorylation [2]. Aminoglycoside phosphotransferases, APHs, catalyze the transfer of a phosphate to a hydroxyl group of the antibiotic. The location of the carbon atom carrying the modified group is specific to each subclass of APH. The most common subclasses are APH(3′) that modify the hydroxyl on the carbon 3 of ring A of the aminoglycoside, and APH(2″) on carbon 2 of the ring C. The name of the subclass of APH is followed by a roman numeral that distinguishes enzymes with different phospho-acceptor and -donor specificity [3]. Finally, isozymes are indicated by a lower case letter.

APH(2″)-IVa confers a high level of resistance in several *Enterococcus* strains towards various aminoglycosides that are prescribed in clinics, and it can use either ATP or GTP as phospho-donor. The first report of the gene encoding this enzyme was by Tsai *et al.* [4]. By the use of an enzyme coupled assay, Toth *et al.* showed that APH(2″)-IVa can phosphorylate all 4,6-disubstituted aminoglycosides, but not the 4,5-disubstituted ones [3]. In a subsequent paper, Toth *et al.* reported the steady state parameters, *k*_cat_ and *K*_m_, of this enzyme with several aminoglycosides and they solved the crystal structure of the apoprotein [5]. They attempted to explain its aminoglycoside specificity by a molecular modeling study that involved the homologous APH(2″)-IIa. One year later, Shi *et al.* determined the crystal structures of APH(2″)-IVa complexed with tobramycin or kanamycin A [6].

Here, we obtained information on the thermodynamics of binding of different aminoglycosides to APH(2″)-IVa by isothermal titration calorimetry. We used crystal structures, complemented with surface potential calculations and molecular dynamics simulation of aminoglycoside release, to describe important interactions between the antibiotics and the enzyme.

To date, there is little information on the reaction pathway of APH(2″)-IVa, apart from the studies of Toth *et al.* [5] who showed that the reaction of APH(2″)-IVa proceeds by a Bi–Bi mechanism in which aminoglycoside and ATP or GTP bind randomly. To fully understand the mechanism of action of an enzyme, one must obtain information on the nature and the rate of interconversion of the intermediates that make up its reaction pathway. This can hardly be obtained from steady state and equilibrium studies alone — it requires transient kinetics [7]. Here, we used a direct quench–flow method, allowing both steady state and transient kinetic measurements, to obtain the time course of total ADP production (APH-bound plus free). Combining this method with free ADP time course measurements using a fluorescent biosensor in a stopped–flow apparatus, has allowed us to determine the nature of the rate-limiting step of the reaction.

## 2 Materials and methods

### 2.1 Chemicals

All aminoglycosides, ATP and other chemicals were obtained from Sigma-Aldrich at the highest purity grade. For control experiments, the sulfate ions contained in the commercialized aminoglycoside powders were removed by Ba(OH)_2_ treatment as described elsewhere [8]. However, the presence of sulfate did not affect the thermodynamic and kinetic parameters. Therefore, sulfate was not removed for subsequent experiments. Equimolar concentrations of MgCl_2_ were added to ATP stock solutions. In the text, unless otherwise stated, ATP refers to MgATP. Aminoglycoside and ATP stock solutions were prepared in 50 mM Tris–HCl, 40 mM KCl and 1 mM free MgCl_2_ and were stored at − 20 °C after adjusting pH to 7.5.

### 2.2 Protein purification

Recombinant APH(2″)-IVa from *Enterococcus casseliflavus* was produced in *Escherichia coli* BL21 (DE3) transformed with pET15b plasmid encoding for APH(2″)-IVa with a 6His-tag in N-terminal. *aph*(*2*″)*-IVa* gene was the generous gift of Professor Vakulenko, Notre Dame, USA.

Two liters of Luria Broth media, LB, supplemented with 0.1 mg/mL of Ampicillin, was inoculated at a final OD_600 nm_ of 0.1 with a 100 mL overnight starting culture. Cells were grown at 37 °C with an agitation of 180 rpm. Protein expression was induced at the late mid log phase (OD_600 nm_ = 0.9) with 1 mM final concentration of IPTG. After an overnight incubation at 20 °C, cells were harvested by centrifugation and the pellet was solubilized in 40 mL of lysis buffer (50 mM NaH_2_PO_4_ at pH 8.0, 300 mM NaCl, 10 mM imidazole, 1 mM DTT and 1 tablet of complete™ EDTA-free protease inhibitor). Bacteria were lysed on ice by sonication. Cell debris were removed by centrifugation and ultracentrifugation steps before loading the supernatant on a HisTrap FF 5 mL column in an Aktä Purifier system (GE Healthcare). The enzyme was eluted from the column with lysis buffer complemented with a linear gradient up to 500 mM of imidazole. Pure fractions were pooled and the protein was concentrated to 30–50 mg/mL on a 30 kDa Centricon® device (Millipore). The elution buffer was exchanged by a storage buffer containing 50 mM Hepes at pH 7.5, 50% glycerol and 1 mM DTT. The protein was stored at − 20 °C. Typical production yield was 80 mg/L of culture.

To improve protein purity for the ITC experiments and crystallogenesis, a supplementary gel filtration step was carried out on a HiLoad 16/60 Superdex 75 column (GE Healthcare) in either 50 mM Tris–HCl pH 7.5, 40 mM KCl, 1 mM MgCl_2_ for ITC or in 50 mM Hepes pH 7.5, 10 mM MgCl_2_ for crystallization assays.

The catalytic activity of APH(2″)-IVa was independent of the presence of the 6His-tag, as controlled by comparing the steady state rates obtained with kanamycin A before and after cleavage of the tagged protein with thrombin (data not shown). The 6His-tag has also no effect on the crystallogenesis of the protein. Therefore, in all experiments the 6His-tag was kept at the N-terminal part of the enzyme.

### 2.3 Isothermal titration calorimetry experiments

The enzyme was extensively dialyzed against buffer containing 50 mM Tris–HCl at pH 7.5, 40 mM KCl, 1 mM MgCl_2_ and ligand solutions were prepared at 5 mM in the final dialysate with readjusting pH to 7.5. The enzyme was used at a final concentration of 150 μM. ITC experiments were carried out at 25 °C in a VP-ITC calorimeter (MicroCal, GE Healthcare). Titrations consisted of 40 injections of 5 μL of ligand every 300 s. The cell stirring speed was 300 rpm. To obtain reliable dissociation constants, the *c*-value, a unitless parameter obtained by the multiplication of the association constant and the total concentration of ligand binding sites, was kept between 1.8 and 100 for all titrations. Control runs were performed by titrating ligands to buffer, and the resulting background signal was subtracted from the corresponding experimental data. Experiments were carried out at 1 mM free Mg^2 +^.

Binding and thermodynamic parameters *K*_a_ (association constant), Δ*H* (enthalpy change) and stoichiometry were obtained by nonlinear least-squares fitting of experimental data using a single-site binding model of the Origin software package (version 5.0) provided with the instrument. The free energy of binding (Δ*G*) and entropy change at 25 °C (Δ*S*) were obtained using the following equations:

(1) $$\Delta G=–\mathrm{RT}\ln\left(K_{a}\right)$$

(2) ΔG=ΔH–TΔS

The experiments were performed in triplicate (except with G418 and kanamycin B, in duplicate) and the error values given in the tables are standard deviations.

### 2.4 Electrostatic potential and free energy calculations

Electrostatic potential surfaces were computed with the APBS program [9]. The charges and radius of all atoms were calculated using PDB2PQR software [10]. The linearized traditional Poisson–Boltzmann equation was solved with APBS using a cubic spline charge discretization and with dielectric constants of 2.0 for the solute and 78 for the solvent at 300 K. Electrostatic potential is represented by a positive and a negative isosurface at ± 10 *K*_b_T/e (*K*_b_, Boltzmann's constant; T, temperature and e, charge of an electron).

All molecular dynamics simulations were performed using the NAMD 2.10 software [11] in the isobaric–isothermal ensemble. The pressure (1 atm) and temperature (300 K) were kept constant using Langevin dynamics and Nosé–Hoover Langevin piston [12], [13]. All protein atoms were described by the CHARMM27 force field [14] while the aminoglycosides were parameterized using the paratool plugin implemented in VMD [15] by assigning both the atomic partial charges computed with Gaussian (by fitting the electrostatic potential surface, ESP) and atom types from CHARMM27. The crystal structures containing either kanamycin A (PDB: 4DFB) or tobramycin (PDB: 3SG8) were immersed in a rectangular water box (TIP3 model) with a 12 Å thickness and neutralized with three or four Na^+^ ions. As the crystal structure of APH(2″)-IVa·sisomicin was not fully solved or unsatisfactory, the complex was obtained by docking (GOLD 5.2 program, CCDC Software Limited) into the chain B of the 4DFB crystal structure. Kanamycin was first removed and used as a center for the search of binding modes (docking poses) of sisomicin by applying 50 runs of genetic algorithms. Solutions were classified according to their scores using the goldscore function. This complex was immersed in a water box as the two other aminoglycosides. The solvated systems were replicated in each direction using periodic boundary conditions. The short-range Lennard–Jones potential was smoothly truncated from 10 to 12 Å and the PME (Particle Mesh Ewald) algorithm [16] was used to calculate long-range electrostatics with a grid spacing of 1 Å. The potential energy of the molecular systems was minimized for 50,000 steps of conjugate gradient (time step of 1 fs). After a gradual heating from 0 to 300 K, the two systems were further equilibrated for 100,000 steps. To explore the “unbinding” of the aminoglycoside from the APH binding site, a reaction coordinate was defined as the distance separating the center of mass of each entity (antibiotic and protein). A biased force was applied using Colvars module of NAMD in order to estimate the free energy change along the reaction coordinates using the ABF method [17]. The distance separating the two centers of mass was initially 7.5 Å and forced to reach 27.5 Å. A width of 0.1 Å was selected using four short window (5 Å) simulations (6 ns each) and the final potential of mean force (PMF) was reconstructed from these separate windows. Simulations were carried out on IBM blade cluster and trajectories were analyzed using VMD.

### 2.5 Crystal structure determination

Crystals of binary complexes of APH(2″)-IVa were grown at 18 °C using similar conditions to those previously described by Toth *et al.* [18]. Co-crystallization of the enzyme with either sisomicin or G418 (Geneticin) was carried out by mixing 1 μL of protein (6 mg/mL^− 1^) and aminoglycoside (2.5 mM) in 50 mM Hepes pH 7.5, 10 mM MgCl_2_ with 1 μL of reservoir solution composed of 12% PEG3350 (*w*/*v*), 50–75 mM ammonium citrate at pH 7.4 to 7.9.

Crystals of binary complex APH(2″)-IVa·sisomicin were grown by the hanging drop vapor diffusion method over a reservoir of 500 μL. After 2–3 days, crystals with approximate dimensions of 300 × 100 × 50 μm were observed. They were briefly immersed in the reservoir solution supplemented with 15% of glycerol as cryoprotectant before being flash-cooled in liquid nitrogen. Data were collected under cryogenic conditions on a Pilatus 6M-F detector at ESRF (Grenoble, France) on beamline ID23-1. Crystals of binary complex APH(2″)-IVa·G418 were grown by the sitting drop vapor diffusion method over a reservoir of 40 μL. The approximate size of crystals was 100 × 50 × 50 μm. Data collection was carried out *in situ* as described by Gelin *et al.* [19] in a 96-well CrystalQuickTM X plate (Greiner BioOne) on beamline BM30A-FIP at the ESRF, equipped with an ADSC Q315r CCD detector. Only the density obtained in chain A was sufficient enough to build G418.

Both structures were refined by molecular replacement using chain A of the homologous APH(2″)-IVa·ADP complex (PDB: 4N57) after removal of the ligand as search model. Data were processed and scaled with XDS [20] and SCALA [21]. The atomic models were rebuilt in Coot [22] and refined using Refmac [23] with 10 sub-segments per protein chain of torsion–libration–screw from the TLSMD server [24]. G418 and sisomicin restraints were generated with the PRODRG2 server [25]. The 2*F*_o_ − *F*_c_ omit maps were calculated with AutoBuild from the PHENIX package [26] after omitting the aminoglycoside molecules present in the asymmetric unit. Data collection and refinement statistics are shown in Table 1 and the structures were deposited in the protein databank (PDB: 5C4K and PDB: 5C4L). The B-factor values for G418 were large which may be explained by the high flexibility of the aminoglycoside in the binding site.

The structures were analyzed using the PyMOL Molecular Graphics System (version 1.3, Schrödinger, LLC). The rmsd between non-hydrogen atoms of different ligands was calculated using the pair fit command.

### 2.6 Kinetic experiments

Unless otherwise stated, kinetic experiments were carried out in a buffer that contained 50 mM Tris, 40 mM KCl, 1 mM free MgCl_2_ adjusted to pH 7.5 with HCl at 25 °C. The initial concentrations of aminoglycoside and ATP are indicated in the figure legends. In the case of experiments with CaATP, the buffer was MgCl_2_-free.

Steady state kinetics were carried out in thermostatically controlled beakers as already described [27]. Unless otherwise stated, the enzyme concentration was 0.5 μM in the presence of various concentrations of aminoglycoside (5 to 100 μM). With amikacin and paromomycin, the absence of activity was confirmed at 5 μM APH(2″)-IVa. Reactions were initiated by adding a saturating concentration of ATP (2.5 mM) in a thermostatically controlled beaker containing APH(2″)-IVa and aminoglycoside. At regular intervals, a volume of 80 μL of the reaction mixture was sampled from the beaker and mixed in a tube containing 40 μL of perchloric acid at 10%. The precipitated protein was then removed by centrifugation at 4 °C, 20 min at 19,000 *g*. ADP and ATP were separated by HPLC (Waters) on an anion-exchange column (SAX-Partisphere, AIT France). The mobile phase was 200 mM ammonium phosphate–HCl buffer at pH 5.5 and 10% acetonitrile. A 100 μL volume of the quenched reaction mixture was diluted in 900 μL of buffer containing the mobile phase supplemented with 5 N KOH to readjust the pH to 5.5. Quantification of ADP and ATP was obtained by integrating HPLC peaks at 259 nm and their concentrations were reported relative to the enzyme concentration. Concentrations (in the quenched reaction mixtures) between 0.5 and 500 μM of ADP or ATP gave a linear and equivalent OD_259_ response. For each injected sample, the concentration of ADP was calculated using the following equation, where [ATP]_i_ is the initial ATP concentration used in the experiment:

(3) $$\left[\mathrm{ADP}\right]=\left(\mathrm{ADP}\;\text{peak}\;\text{area}\times {\left[\mathrm{ATP}\right]}_{i}\right)/\left(\mathrm{ADP}\;\text{peak}\;\text{area}+\mathrm{ATP}\;\text{peak}\;\text{area}\right)$$

Transient kinetic experiments were carried out at 25 °C in a QFM-400 quench–flow (Bio-Logic, France) or in a SF-61 DX2 stopped–flow (TgK Scientific, UK). In the quench–flow method, APH(2″)-IVa, pre-incubated with the aminoglycoside, was mixed with ATP in the apparatus. The mixtures were aged for specific times (from 8 ms to several seconds), quenched in 10% perchloric acid and the ADP and ATP measured by HPLC, as described above. Because perchloric acid dissociates all non-covalent complexes, by this method, concentrations in the reaction mixtures of *total* ADP, that is to say enzyme-bound ADP plus free ADP, are obtained [27]. In the stopped–flow experiments, a free ADP fluorescent biosensor, MDCC-ParM [28], was included in the ATP solution. By this method, concentrations in the reaction mixtures of *free* ADP only are obtained. Experiments were carried out as previously described for APH(3′)-IIIa [27].

## 3 Results

**Fig. 1:**
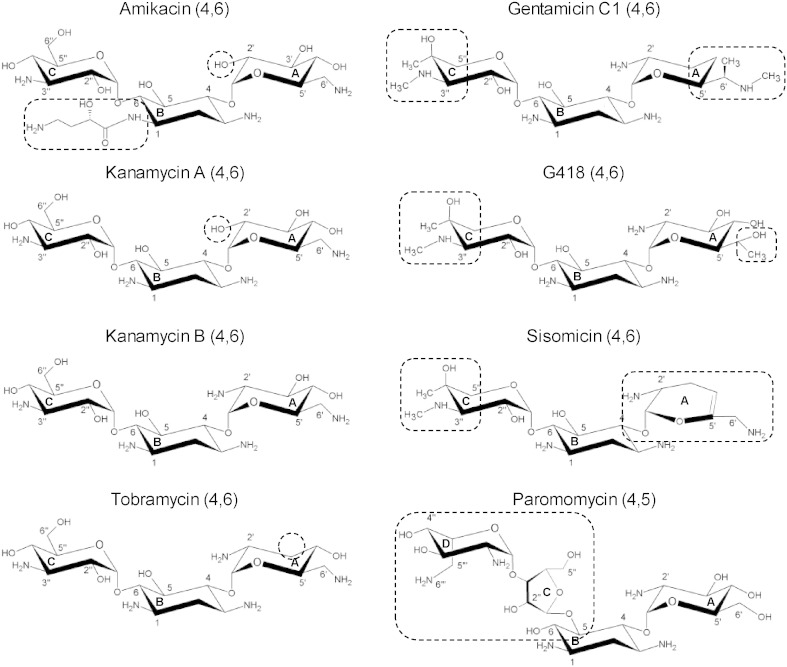
Structure of the aminoglycosides investigated in this study. Substitutions on the 2-deoxystreptamine scaffold (4,5) or (4,6) are indicated. Dashed lines represent structural elements that are discussed in the text.

The structures of the eight aminoglycosides studied here are given in Fig. 1.

### 3.1 Thermodynamic studies of aminoglycosides binding to APH(2″)-IVa

By isothermal titration calorimetry (ITC), we determined the thermodynamic parameters, and consequently the binding affinities, of aminoglycoside binding to APH(2″)-IVa. Although ITC has been used extensively to characterize aminoglycoside binding to APH(3′)-IIIa [29], [30], [31], [32] or to other aminoglycoside modifying enzymes [33], [34], [35], [36], [37], [38], [39], this work brings the first data for APH(2″)-IVa. Fig. S1 shows the binding isotherms and parameters obtained with APH(2″)-IVa and eight aminoglycosides, fitted with a single site model. Thermodynamic parameter values obtained with 4,6- or 4,5-disubstituted aminoglycosides are in Table 2. For all the aminoglycosides, the free energy changes, Δ*G*, were negative upon binding to APH(2″)-IVa, indicating that the formation of APH(2″)-IVa ∙ aminoglycoside complexes is favorable, even with 4,5-disubstituted aminoglycosides that are not substrates of this enzyme ([5] and confirmed below). Values of Δ*G* range from − 7.8 to − 5.6 kcal mol^− 1^, corresponding to *K*_d_ of 2.3 to 82.2 μM.

**Fig. 2:**
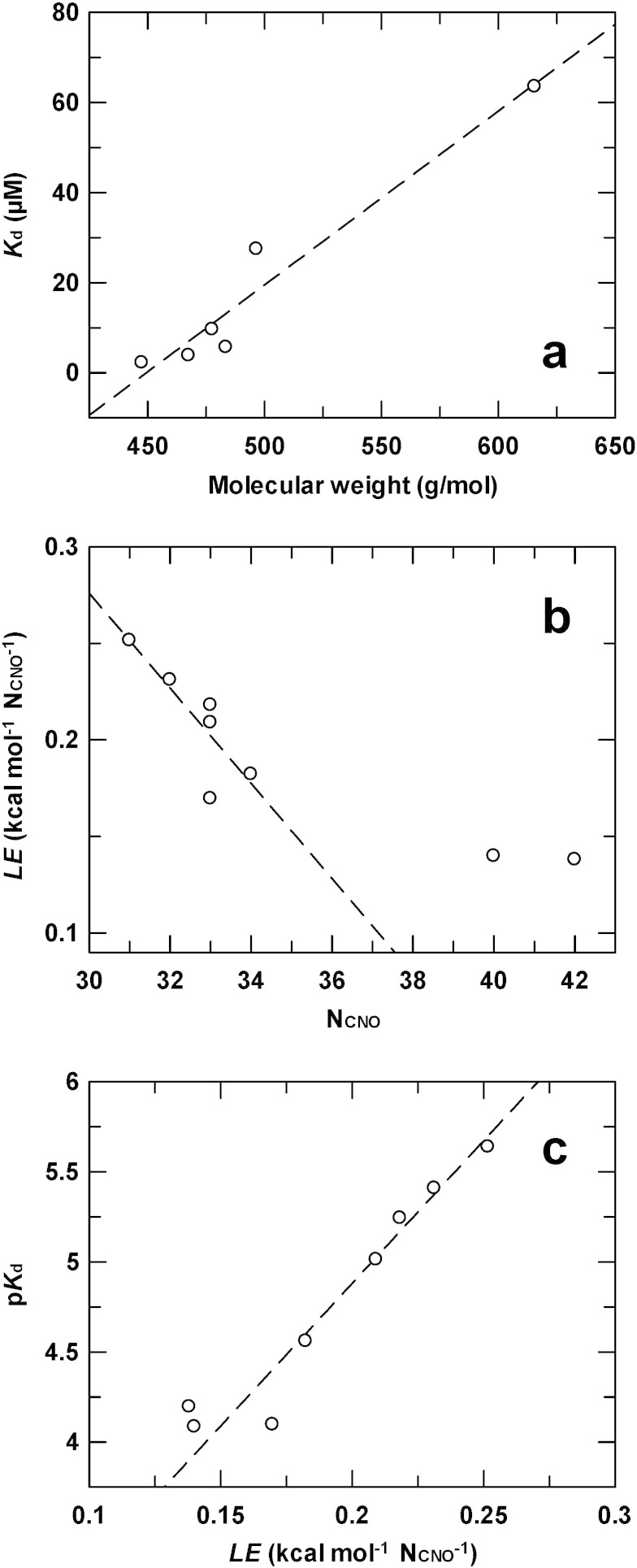
Relationship between the size of aminoglycosides and their affinity for APH(2″)-IVa determined by ITC. (a) Representation of the *K*_d_ value as a function of the molecular weight of aminoglycosides. Data for kanamycin A and amikacin have been excluded from analysis. (b) Representation of the ligand efficiency, *LE*, as a function of the number of non-hydrogen atoms of aminoglycoside (N_CNO_). (c) Dependence of the logarithm of *K*_d_ (p*K*_d_) on *LE*. Dashed lines indicate the general tendencies.

Interestingly, there is a correlation between affinity and aminoglycoside molecular weight as shown in Fig. 2a. Excluding kanamycin A and amikacin, the two aminoglycosides having a hydroxyl instead of an amino group in C2′ position (discussed later) that showed the lowest affinities, the smallest aminoglycosides exhibited the tightest binding to APH(2″)-IVa. Another way to illustrate this observation is to exploit ligand efficiency, *LE*, a guide commonly used in drug discovery and lead optimization [40], [41]. Ligand efficiency is defined as the ratio of − Δ*G* to the number of non-hydrogen atoms of the ligand (N_CNO_). As shown in Fig. 2b, ligand efficiency linearly depends on N_CNO_ with the largest aminoglycosides exhibiting the smallest ligand efficiencies. Consistently, there is a logarithmic dependence of *K*_d_ on ligand efficiency (Fig. 2c).

Does the presence of nucleotide affect APH(2″)-IVa affinity for aminoglycosides? With APH(3′)-IIIa, the formation of binary enzyme ∙ aminoglycoside and ternary enzyme ∙ ATP ∙ aminoglycoside complexes could be compared by the use of CaATP, because with it the enzyme is inactive [30]. It was not possible to assess this question with APH(2″)-IVa as CaATP is a substrate of this enzyme (Fig. 5a, see below). Instead, we used MgADP or MgAMP-PNP and showed that the pre-incubation of the enzyme with the nucleotide has no significant effect on its affinity for aminoglycoside or on binding thermodynamic properties. For example, with kanamycin A, the *K*_d_ values were 87 ± 8 μM in the presence of 870 μM MgADP, 103 ± 12 μM in the presence of 1 mM MgAMP-PNP and 80 ± 6 μM in the absence of nucleotide.

### 3.2 Electrostatic potential and free energy change calculations

**Fig. 3:**
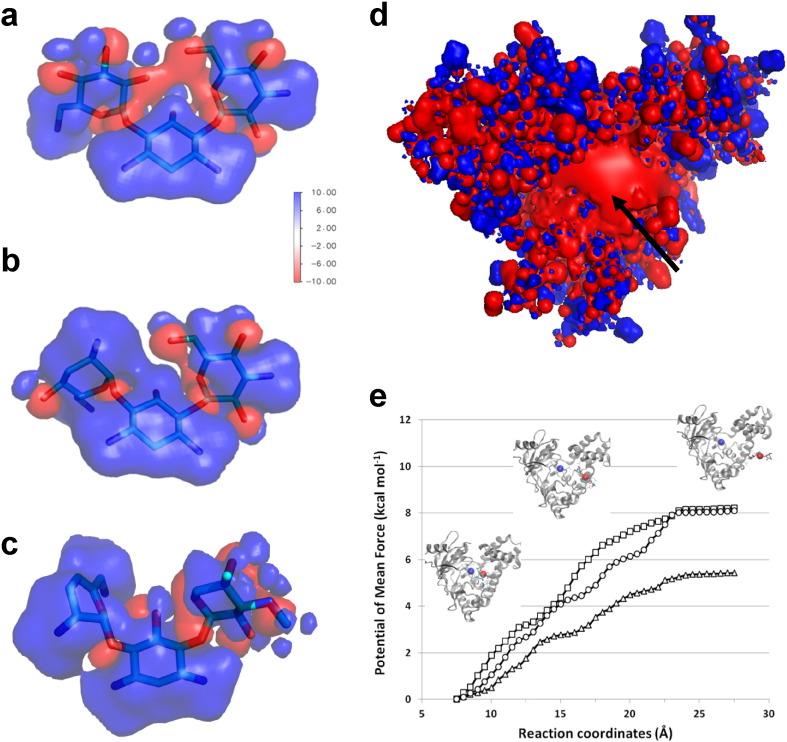
Electrostatic potential surface representation of kanamycin A (a), tobramycin (b) and sisomicin (c) computed using Adaptive Poisson–Boltzmann Solver (probe radius 1.4 Å). The blue and red surfaces show the positive and negative isosurfaces of the electrostatic potential at ± 10 kT/e, respectively. (d) The same representation as in previous panels but for APH(2″)-IVa (PDB: 4DFB in the absence of kanamycin A). (e) Potential of mean force (PMF) computed from dynamics simulations using the ABF method for unbinding kanamycin A (∆), tobramycin (□) and sisomicin (○). The reaction coordinate was defined by the distance separating the two centers of mass (APH and aminoglycoside). Insert: snapshots showing the kanamycin unbinding as a function of time during the simulation (each center of mass is depicted as a red or blue sphere).

In order to determine the nature of the preponderant interactions governing the aminoglycoside binding to APH, the electrostatic potential surface of kanamycin A, tobramycin and sisomicin was computed (Fig. 3a to c) and showed obvious differences in their charge distribution. Indeed, for tobramycin and sisomicin, the supplementary positively charged amino group in 2′ position induces a very large positive surface compared to that of kanamycin A. This should be highly favorable to strong binding to APH(2″)-IVa, since the aminoglycoside binding site is fully negatively charged (Fig. 3d).

The binding free energy measured by ITC for the different aminoglycosides showed very small differences. Therefore, in order to explore thoroughly these small energy barriers, molecular dynamics simulations using the ABF method were carried out on several complexes. Free energy calculations and the resulting PMF (estimation of the energy required for ligand release) are shown in Fig. 3e.

As expected, the force required to unbind the aminoglycoside was similar for the three aminoglycosides tested. However, one can notice a higher PMF obtained in the case of tobramycin and sisomicin as compared to kanamycin A. These differences in terms of free energy are in good agreement with the values obtained by ITC. Indeed, the Δ*G* values of − 5.6, − 7.4 and − 7.8 kcal mol^− 1^ measured by ITC for kanamycin A, tobramycin and sisomicin were comparable to the PMF values which were 5.4, 8.2 and 8.1 kcal mol^− 1^, respectively (values at the end of the simulation, with opposite sign to Δ*G* as unbinding is measured here). Comparison of such theoretical values should be taken with care, as the full binding process is not computed, but instead just the release of the aminoglycoside.

Interestingly, the dissociation of the aminoglycoside seems to be governed by the NH_3_^+^ groups that link successively numerous Asp or Glu residues present at the periphery of the aminoglycoside binding site. This is shown by the different phases punctuating the PMF curves (Fig. 3e) and this may reflect the entry or the exit pathway of the aminoglycoside.

### 3.3 Structure determination of APH(2″)-IVa ∙ aminoglycoside binary complexes

In order to characterize interactions between enzyme and ligands, we determined the crystal structures of APH(2″)-IVa bound to aminoglycosides with different affinities.

A crystal structure of APH(2″)-IVa in complex with sisomicin, that has the lowest *K*_d_ of the eight aminoglycosides tested here, has been solved at a resolution of 2.35 Å (Fig. S2). However, the binding position of the aminoglycoside in chain B is probably induced by the crystal packing. Indeed, sisomicin is stabilized by an interaction with the backbone of Ser136, which belongs to a symmetric protein of the unit cell. This feature shifts the position of the aminoglycoside from its presumed physiologically relevant orientation. In chain A, the electron density obtained permits only to build the ring A of sisomicin. The rest of the molecule is probably too flexible to be visualized. In both chains, sisomicin is stabilized by salt bridges occurring between the side chains of Glu235 and Glu268 and the 6′-NH_3_^+^ of the aminoglycoside and by hydrophobic interaction between Trp271 and ring A.

**Fig. 4:**
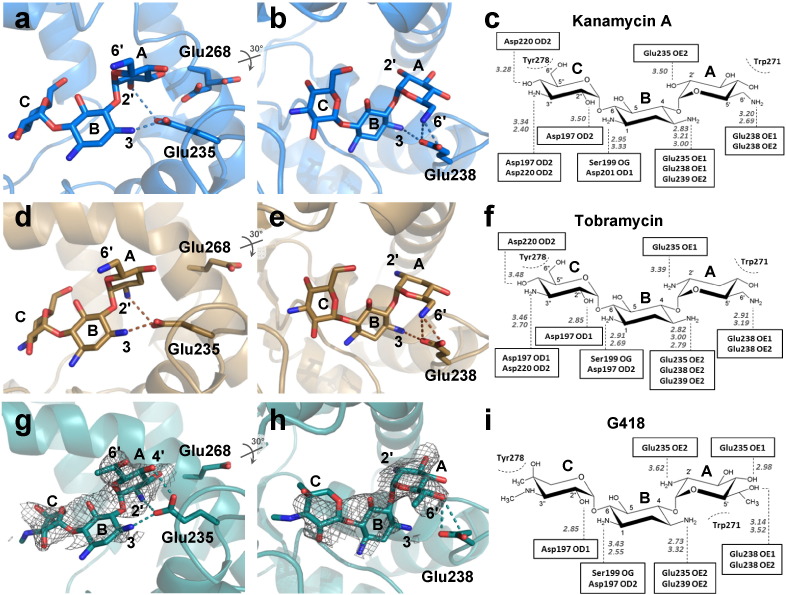
Comparison of tobramycin, kanamycin A and G418 binding to APH(2″)-IVa. Crystal structures of APH(2″)-IVa in complex with (a, b) kanamycin A, (d, e) tobramycin or (g, h) G418 with the ligand contoured with the 2*F*_o_ − *F*_c_ omit map (gray) at a sigma level of 1. Respective details of interactions are shown for kanamycin A (c), tobramycin (f) and G418 (i). The left panels show the interactions of Glu235 with the aminoglycosides. Middle panels show interactions of Glu238. The aminoglycosides are figured in stick representation and ring nomenclature is shown. The major interactions observed between the three aminoglycosides and the APH(2″)-IVa residues are shown as dashed lines.

We solved the structure at a resolution of 3.05 Å of APH(2″)-IVa in complex with G418 (geneticin), that has an intermediate affinity for APH(2″)-IVa. Comparison of the crystal structures of binary complexes with tobramycin, kanamycin A and G418 (PDB: 3SG8, 4DFB and 5C4K, respectively) shows that the position of the three aminoglycosides is highly similar with an rmsd (between ligands non-hydrogen atoms) of 0.59 Å between kanamycin A and G418 and of 0.36 Å between tobramycin and G418 (Fig. 4).

As already observed in the previous published structures, Glu235 stabilizes rings A and B of the aminoglycoside (Fig. 4a, d, g). Interestingly, the interaction between Glu238 and the ring A of aminoglycoside is maintained even if the substituent in the 6′-position of G418 is different (Fig. 4b, e, h). However, its interaction with ring B is, in turn, abolished. Similar to kanamycin A and tobramycin structures, ring C is maintained by an interaction with Asp197. In general, the APH(2″)-IVa ∙ G418 complex is characterized by less interactions than the binary complex with tobramycin, which may be at the origin of the weaker affinity of G418 compared to tobramycin.

### 3.4 Steady state and transient kinetics by direct assessment of ADP production

In order to see if the differences in the binding affinity of aminoglycosides affect the enzymatic reaction rate constants, we carried out steady state and transient kinetic experiments. Here, we used a direct method for separating and quantifying ADP and ATP by HPLC in APH reaction mixtures that had been quenched at different times in acid. With this method, concentrations of ADP down to 0.5 μM can be measured.

**Fig. 5:**
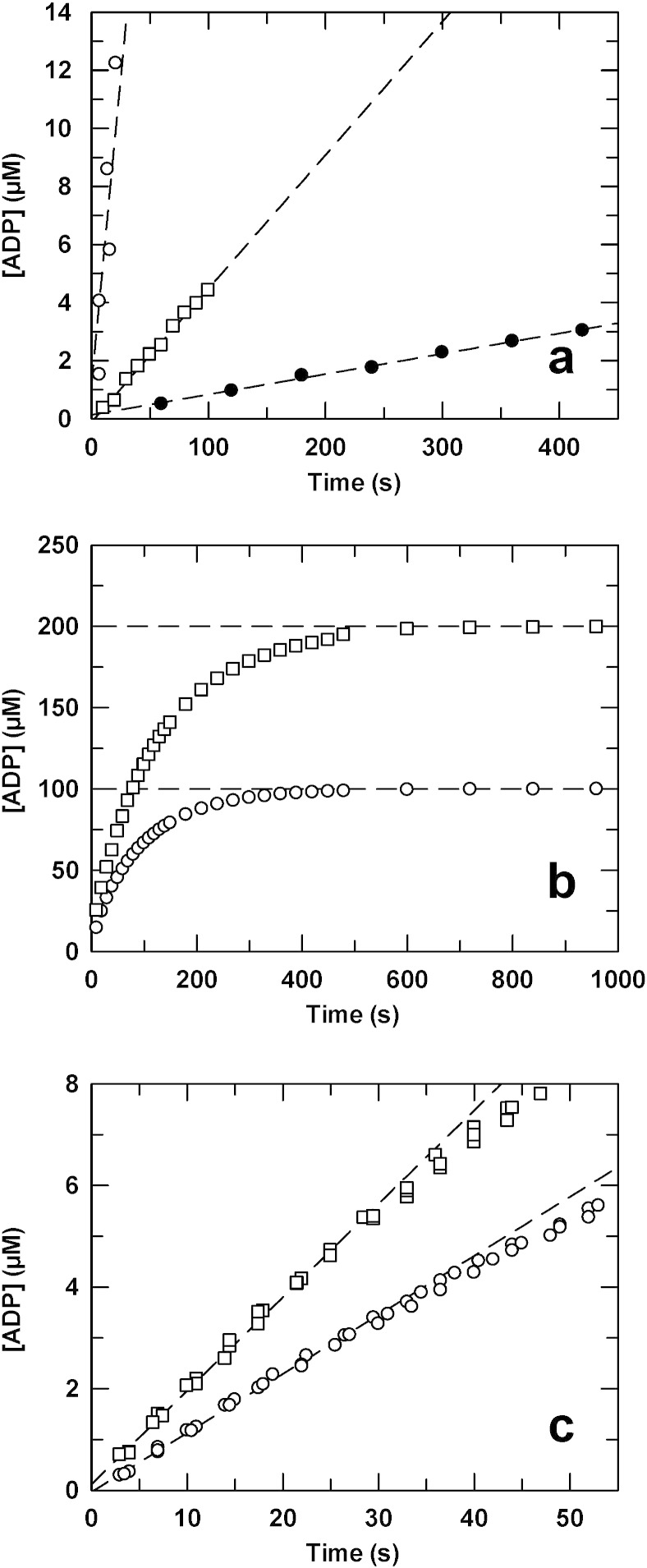
Time courses of ADP formation by the quenching method and HPLC analysis. (a) Time courses of MgATPase (●, *k*_ss_ = 0.014 s^− 1^), CaATP phosphotransferase (□, *k*_ss_ = 0.092 s^− 1^) and MgATP phosphotransferase (○, *k*_ss_ = 0.84 s^− 1^) activities. APH(2″)-IVa concentration was 0.5 μM, kanamycin A was 0 or 100 μM, and MgATP or CaATP was 2 mM. (b) Complete time courses for MgATP phosphotransferase activity at 5 μM APH(2″)-IVa, 500 μM kanamycin A and 100 (○) or 200 (□) μM ATP. (c) Initial portions of the time courses showing linearity up to 30 s at 0.5 μM APH(2″)-IVa, 100 μM kanamycin A and 100 (○) or 200 (□) μM MgATP. The experiments were done in triplicate. The steady state rate constants were 0.23 s^− 1^ and 0.37 s^− 1^, respectively.

In addition to its phosphotransferase activity, in the absence of aminoglycoside, APH(2″)-IVa has a low ATPase activity of 0.014 s^− 1^ with 2 mM MgATP (Fig. 5a). Its phosphotransferase activity in the presence of 2 mM CaATP as phospho-donor and kanamycin A as acceptor was low compared to that with MgATP, but not negligible (0.092 and 0.84 s^− 1^, respectively). Typical time courses of the phosphotransferase activity at two different initial concentrations of MgATP are illustrated in Fig. 5b. It is noteworthy that in each case the reaction went to near completion, which is to say that ATP was completely converted into ADP. Steady state rates were obtained from the initial linear portions (Fig. 5c).

The steady state parameters, *k*_cat_ and *K*_m_, obtained with 8 aminoglycosides and with MgATP as phosphate donor, are summarized in Table 3. As can be seen, with the exception of tobramycin, our values are in reasonable agreement with those of Toth *et al.* using the coupled enzyme system [5]. With tobramycin, Toth *et al.* reported a *K*_m_ of 1.5 μM whereas we obtained 13.2 μM. We are unable to explain this difference, but this could potentially be a consequence from using a coupled assay versus a direct method such as the quench–flow assay.

**Fig. 6:**
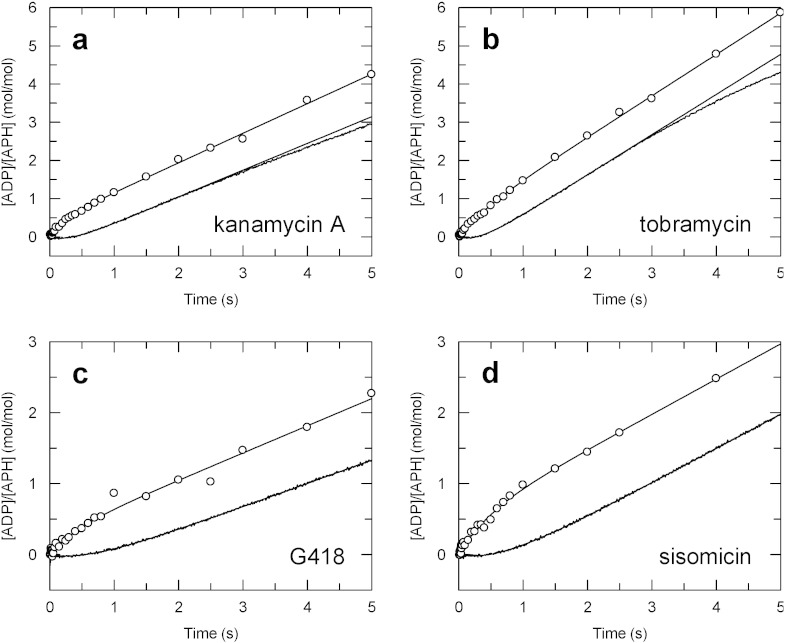
Rapid quench–flow (circles) and stopped–flow (lines) time courses of ADP formation with different aminoglycosides. The quench–flow reaction mixtures contained 5 μM APH(2″)-IVa, 500 μM ATP and 100 μM kanamycin A (a), tobramycin (b), G418 (c) or sisomicin (d). Time courses fitted to a single exponential burst followed by a steady state. In stopped–flow experiments, the reaction mixtures were the same, plus 50 μM MDCC-ParM. Time courses fitted to an initial lag (fitted with a single exponential) followed by a steady state. Values of fitted parameters are given in Table 4.

The variation of the ratio *K*_d_/*K*_m_ between aminoglycosides is striking — from 20 with kanamycin A to 0.3 with tobramycin. Are these different ratios explained by differences in the kinetics of the transient formation of enzyme ∙ ADP complex? Using the same quenching/sampling method and a rapid quench–flow apparatus, we could measure transient kinetics of *total* ADP production catalyzed by APH(2″)-IVa with kanamycin A, tobramycin, G418 or sisomicin and ATP, as illustrated in Fig. 6 (circles).

Except the nature of the antibiotic, the four experiments were carried out under identical conditions. Each time course for the production of *total* ADP (that is to say, free ADP plus enzyme-bound ADP which dissociates with the acid quenching) consists of two phases: a burst of ADP followed by the steady state. These traces suggest that an ADP-containing complex or free ADP is rapidly formed compared to the steady state rate of the reaction.

In parallel, and in the same experimental conditions, we used fluorescence stopped–flow with an ADP biosensor in order to measure the kinetics of *free* ADP production. The reaction was followed by measuring the ADP-induced fluorescence increase of MDCC-ParM as a function of time. Results are shown in Fig. 6 as continuous lines. Interestingly, the time courses of *free* ADP production showed a lag preceding the steady state, the amplitude and rate constant of which are similar to those determined for the burst of *total* phosphate. This suggests that *free* ADP is not formed rapidly compared to the steady state rate of the reaction, but instead an ADP-containing complex — the lag is a reflection of the time needed to build up this intermediate. Values for the amplitudes and rate constants of the burst/lag phases and the steady state rate constants are summarized in Table 4.

The transient kinetic parameters determined here did not show large differences between the aminoglycosides, although there may be a correlation between *k*_cat_ and the transient rate constants (*k*_lag_ and *k*_burst_). Also, transient rate constants were not very large compared to the steady state rate constants. This is especially true for tobramycin with only a 2 to 3-fold difference. Altogether, these results suggest that with all aminoglycosides, even those with a low affinity for the enzyme, the product release is the rate limiting step of the reaction, but the phosphotransfer step, that leads to the production of ADP-containing complex, may also contribute.

## 4 Discussion

### 4.1 Comparison of thermodynamic parameters of different aminoglycoside phosphotransferases

APH(2″)-IVa is specific for 4,6-disubstituted aminoglycosides as phosphate acceptors. In comparison, APH(3′)-IIIa has a broader specificity for antibiotics — it can use 4,5- as well as 4,6-disubstituted aminoglycosides. It is therefore interesting to compare the aminoglycoside affinities of APH(3′)-IIIa and APH(2″)-IVa, two enzymes that catalyze the same reaction but on different positions on the aminoglycoside and with a different substrate specificity. The *K*_d_ values determined here for 8 aminoglycosides with APH(2″)-IVa are comparable to those measured by Özen and Serpersu [30] with APH(3′)-IIIa (*K*_d_ between 0.3 and 92.6 μM). With both phosphotransferases, amikacin shows a much lower affinity relative to other aminoglycosides, probably due to its bulky substituent at position N1.

**Fig. 7:**
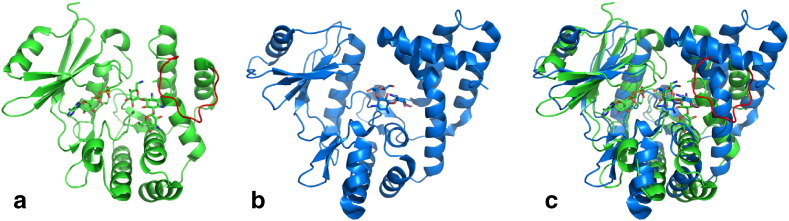
Comparison of aminoglycoside binding site of APH(3′)-IIIa and APH(2″)-IVa. (a, b) Structures of the APH(3′)-IIIa (PDB: 1L8T, green) and the APH(2″)-IVa (PDB: 4DFB, blue), respectively. The loop V154-K166 is colored in red in the 1L8T structure. (c) Superposition of the two structures.

Another difference between these two phosphotransferases is that the affinity for aminoglycosides depends on the antibiotic size with APH(2″)-IVa, but not with APH(3′)-IIIa. With APH(2″)-IVa, this correlation may be the effect of entropic contributions where longer substrates are restricted in degrees of freedom in the binding site, without proper compensation with enthalpic terms, since the additional residues of the substrate are not interacting strongly with the enzyme. This suggests that the binding site on APH(2″)-IVa is less extensible and dynamic than that of APH(3′)-IIIa. Indeed, molecular dynamics simulations [42] showed that a highly flexible loop of APH(3′)-IIIa (V154-K166), positioned over the antibiotic binding pocket and colored in red in Fig. 7a, becomes more constrained in the presence of kanamycin A, with which it interacts. The flexibility of this loop may explain the ability of the APH(3′)-IIIa to accommodate aminoglycosides with various sizes, contrary to the APH(2″)-IVa. Indeed, this loop is not present in the latter (Fig. 7b), which probably limits the binding of 4,5-disubstituted aminoglycosides or, at least, dramatically decreases their affinity for the protein.

However, this alone does not explain the lack of catalytic activity of APH(2″)-IVa with the 4,5-disubstituted aminoglycosides. Instead, unfavorable positioning of the 2″-site may be the reason. This is similar to what is observed with the aminoglycoside nucleotidyltransferase(2″)-Ia, to which neomycins bind tighter but are not substrates of the enzyme. In this case, the 2″-site becomes too distant to the α-phosphate group for a direct nucleophilic attack [35]. Thus, unfavorable orientation of the 2″-site in the more constrained binding site on APH(2″)-IVa may be correlated with its inability to catalyze phosphotransfer with 4,5-disubstituted aminoglycosides.

### 4.2 Comparison of the interactions between APH(2″)-IVa and aminoglycoside in the crystal structures

Five APH(2″)-IVa ∙ aminoglycoside binary complexes have been solved by X-ray crystallography: two in the presence of kanamycin A (PDB: 4DFB [43] and PDB: 3SG9 [6]), one with tobramycin (PDB: 3SG8 [6]), one with G418 (PDB: 5C4K, here) and one with a partially solved sisomicin (PDB: 5C4L, here).

**Fig. 8:**
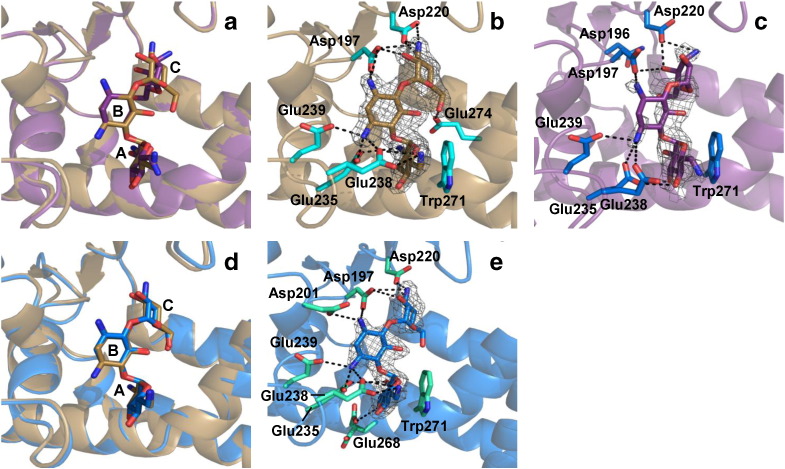
Crystal structures of APH(2″)-IVa ∙ aminoglycoside binary complexes. The enzyme is shown in cartoon representation and the substrate in sticks. (a) Superposition of tobramycin in brown (PDB: 3SG8, chain A) and kanamycin A in purple (PDB: 3SG9, chain A) structures. (b, c) Individual APH(2″)-IVa ∙ tobramycin and APH(2″)-IVa ∙ kanamycin A complexes, respectively. Aminoglycosides are contoured by their 2*F*_o_ − *F*_c_ electron density mesh at 1.0 *σ* (gray). Interacting residues are represented in cyan sticks and interaction highlighted by dashed lines. The electron density maps were from the Electron Density Server (EDS). (d) Superposition of APH(2″)-IVa ∙ tobramycin in brown (3SG8, chain A) and APH(2″)-IVa ∙ kanamycin A in blue (PDB: 4DFB, chain B) structures. (e) 2*F*_o_ − *F*_c_ electron density map obtained in 4DFB.

In the structures determined by Shi *et al.* [6], the superposition of the two different aminoglycosides reveals similar binding positions, except for ring C, coplanar to ring A for kanamycin A and lightly tilted for tobramycin (Fig. 8a). High resolution electron densities were visible for the aminoglycoside in the tobramycin structure (3SG8), see Fig. 8b. In contrast, we found that the density obtained for both chains of APH(2″)-IVa ∙ kanamycin A complex (3SG9) was not sufficient to completely build the ligand (Fig. 8c), resulting in much larger B-factor values than the surrounded residues. Nevertheless, the superposition of the APH(2″)-IVa ∙ tobramycin structure (3SG8) and that with kanamycin A solved by Shakya *et al.* (4DFB) shows identical positioning of aminoglycosides in the binding site (Fig. 8d) and very slight modifications of interacting amino acid side chains. The electron density obtained for 4DFB fully covers the aminoglycoside substrate (Fig. 8e). We consequently decided to use the 4DFB structure for following discussion.

Comparing the APH(2″)-IVa ∙ tobramycin (Fig. 8b) and the APH(2″)-IVa ∙ kanamycin A (Fig. 8e) binary structures, most interactions between the aminoglycoside and protein residues are conserved. Nevertheless, there may be a supplementary H-bond between Glu268 and the 3′-OH of kanamycin A which is not present in the tobramycin structure, not substituted in 3′ (Fig. 8b and e). However, this interaction may be weak because the distance measured between the 3′-O of kanamycin and the δ-O of Glu268 is rather long for a H-bond (3.57 Å) in chain B and it does not exist in the chain A, where Glu268 occupies an alternative position as shown in Fig. 4a. More importantly, the H-bond formed between Glu235 and 2′-OH of kanamycin A is replaced by a salt bridge with 2′-NH_3_^+^ of tobramycin or G418. As a result, the affinity of the apoprotein is 20 times greater for tobramycin than for kanamycin A and for amikacin which also lacks a 2′-NH_3_^+^.

### 4.3 Effect of different substituents of aminoglycosides on APH(2″)-IVa affinity and catalytic activity

In comparison with other aminoglycosides, the different substituents in positions C3″, C4″ and C5″ of gentamicin, G418 and sisomicin do not alter significantly the affinity for APH(2″)-IVa. We note that these three aminoglycosides have the lowest *k*_cat_ and *K*_m_ values of the eight tested. Since they also present modifications on ring A, it is difficult to draw clear conclusions concerning the effect of ring C substituents. However, the crystal structure of the APH(2″)-IVa ∙ G418 complex shows that the hydroxyl and methyl groups on the C6′ of G418 do not create novel interactions with the protein (Fig. 4h and i). The absence of a primary amine on C6′ of ring A seems to affect the binding affinity of aminoglycosides for APH. Indeed, G418 has a weaker affinity for the apoprotein than gentamicin, with a secondary amine at C6′, and even weaker than sisomicin with a primary amine. The crystallographic structure obtained in the presence of sisomicin shows that the aminoglycoside is stabilized by salt bridges between 6′-NH_3_^+^ and Glu235 and Glu268 (Fig. S2). An interaction of 2′-NH_3_^+^ of sisomicin with Asn228 may also play a role. Even if these data are incomplete or biased by the crystal packing, these results highlight the importance of 2′- and 6′-NH_3_^+^ groups in the binding of aminoglycosides, explaining the very high affinities obtained by ITC.

Docking of sisomicin predicts a similar position of binding compared to the crystal structure (Fig. S2e and h). Indeed, the 2′- and 6′-amines are still involved in crucial interactions with Glu235, Glu239 and Glu268. However, the predicted binding mode shows that the 2′-NH_3_^+^ group of sisomicin is lightly shifted towards Glu235, creating more interactions with the glutamate residues. The presence of a double bond which makes the A ring more rigid should drastically reduce the conformational freedom of the ring and therefore may stabilize the binding of the aminoglycoside by favoring a particular ring orientation.

The difference in position 1 of amikacin compared with the other aminoglycosides results in a dramatic lowering of its catalytic efficiency. However, this different substituent does not affect much its binding to the apoenzyme as its *K*_d_ is very similar to that of kanamycin A (82.2 μM and 79.9 μM, respectively). The bulky group in the C1 position of amikacin may induce steric hindrance which could be deleterious for the phosphotransfer on the 2″-OH.

### 4.4 Interpretation of APH(2″)-IVa transient kinetic data

**Scheme 1:**
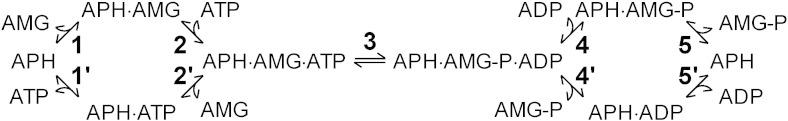
Random bi–bi mechanism of the phosphotransfer reaction catalyzed by APH(2″)-IVa.

As a first approximation, we interpret our data via Scheme 1 in which APH represents APH(2″)-IVa, AMG aminoglycoside and AMG-P the corresponding 2″-phosphorylated aminoglycoside.

The steps 1 to 5 are described by the forward rate constants *k*_+ i_ and the backward rate constants *k*_*−* i_. For simplicity, in Scheme 1 substrate binding and product release steps are shown as single steps, but they are most probably composed of two steps: a rapid equilibrium leading to the formation of the collision complex, followed by a conformational change. Toth *et al.* [5] proposed that APH(2″)-IVa catalyzes the phosphotransfer by a random mechanism, that cannot exclude the possibility that the binding of one substrate affects the binding of the other (that is to say *K*_1_ ≠ *K*_2_′ and *K*_1_′ ≠ *K*_2_). Isothermal titration calorimetry experiments carried out here gave information on step 1. The *K*_d_ for aminoglycoside represents the equilibrium constant of aminoglycoside binding step, that is *K*_1_ = *k*_− 1_/*k*_+ 1_. In our experimental conditions, we measured a 20-fold larger *K*_d_ for kanamycin A compared to tobramycin. Now, unexpectedly, under catalytic conditions, that is in the presence of ATP, the enzyme had a 3.3-fold lower *K*_m_ for kanamycin A compared with tobramycin. Segel proposed that, if the ratio *K*_m_/*K*_d_ for a given substrate is < 1, there is substrate synergy by which the binding of one substrate increased the affinity for the second [44]. On the other hand, if the ratio is > 1, there is substrate antagonism (for a recent discussion, see [45]). With the caveat that a *K*_m_ is not a simple dissociation constant for the formation of a ternary enzyme complex, this suggests that the presence of ATP may have different effects on the interactions of kanamycin A and tobramycin with the enzyme, although we showed that ADP or AMP-PNP did not.

Alternatively, it could be that for efficient catalysis, the aminoglycoside must not be too strongly bound to the phosphotransferase to allow the rapid release of the phosphorylated antibiotic. Thus, if the phosphorylated aminoglycoside interacts tightly with the enzyme, it could slow down its release kinetics. A slow release would manifest itself by the accumulation of intermediates that involve the products. We confirmed this by transient kinetics: starting with APH ∙ AMG plus ATP, the time course of *total* ADP formation was biphasic with a rapid burst of ADP preceding the steady state rate whereas that of *free* ADP was biphasic with a lag instead of the burst. These observations suggest that intermediates containing ADP (APH ∙ AMG-P ∙ ADP and/or APH ∙ ADP) accumulate in the steady state. Therefore, we can conclude that with APH(2″)-IVa, steps 2 and 3 are fast compared to step 4 or 5′. It implies that ADP release, step 4 or 5′, is rate-limiting. We note that with three different APH(3′), a product release step is also rate limiting [27], [46], [47].

However, the observation that the transient rate constants measured here were not much larger than steady state rate constants, suggests that step 3 may not be much faster than the product release steps. To evaluate the relative contribution of each step on the transient and steady state kinetics, we intend to study the dependence of the transient amplitudes and rate constants as a function of the aminoglycoside or ATP concentrations, and starting from the apoenzyme or binary complexes.

Shi and Berghuis published crystal structures of APH(2″)-IVa in complex with adenosine and guanosine that provide explanation for the peculiar dual nucleotide specificity of this enzyme [48]. Therefore, it would be interesting to compare the transient kinetic rate constants obtained using MgATP or MgGTP as phosphate donor, to determine if the phosphotransfer step is similarly fast with the two nucleotides.

## 5 Conclusions

Altogether, our study strongly suggests that two primary amino groups are required for a tight binding of the aminoglycoside to APH(2″)-IVa. The presence of both amino groups in position 2′ and 6′ leads to strong affinities by interacting with Glu235 and Glu238, respectively. In addition, it must be highlighted that the binding of the aminoglycoside to APH is mainly driven by electrostatic interactions. However, a better affinity is not correlated with a better catalytic efficiency.

We show that the reaction catalyzed by APH(2″)-IVa is kinetically limited by the release of product. These results may provide further insights into the mechanisms by which bacteria evolved to combat aminoglycoside antibiotics and may aid in the design of specific drugs that overcome this resistance.

The following are the supplementary data related to this article.

Fig. S1 Binding isotherms and fits obtained with (a) kanamycin A, (b) kanamycin B, (c) sisomicin, (d) tobramycin, (e) paromomycin, (f) amikacin, (g) gentamicin and (h) G418. (i) Thermodynamic parameters obtained: ∆* G* (gray), ∆* H* (dashed) and − T ∆* S* (white). Data are shown as mean ± standard deviation from 2–3 independent repetitions. From left to right, aminoglycosides are ranked from the highest to the lowest absolute value of ∆* G*.

Fig. S2 Structural analysis of hypothetical sisomicin binding site. Panels a and d show the 2*F*_o_ − *F*_c_ omit maps contoured at 1 σ (gray) obtained by X-ray crystallography for chain A and chain B, respectively. The density in chain A (a) only permits the positioning of the ring A of sisomicin. Stabilizing interactions are detailed (b, c). The density in chain B (d) shows a different packing-induced binding position of sisomicin (e, f) which forms a stabilizing interaction with the backbone of Ser136 belonging to a symmetric protein of the unit cell. (g) Predicted binding mode (docking pose) of sisomicin in the 4DFB protein after removal of kanamycin A. Main interactions are figured (h) and detailed (i).

## Transparency document

Transparency document.

## Figures and Tables

**Table 1:** Data collection and refinement statistics of APH(2″)-IVa ∙ aminoglycoside complexes. Values in parentheses refer to reflections in the highest-resolution shell. Ramachandran statistics are from Coot.

Aminoglycosides	G418	Sisomicin
PDB code	5C4K	5C4L
Data collection		
Temperature (K)	298	100
Space group	P 1 21 1	P 1 21 1
Cell dimensions		
a, b, c (Å)	78.70, 64.94, 78.87	74.21, 65.11, 78.89
α, β, γ (°)	90.00, 90.73, 90.00	90.00, 91.16, 90.00
Resolution (Å)	42.44–3.05 (3.16–3.05)	37.10–2.35 (2.43–2.35)
*R*_*sym*_ or *R*_*merge*_^a^	0.067 (0.318)	0.034 (0.230)
*I*/*σI*	5.5 (1.9)	8.9 (2.5)
Completeness (%)	90.0 (94.8)	94.7 (96.6)
Redundancy	1.6 (1.5)	1.9 (1.9)
Refinement		
Resolution (Å)	42.44–3.05	37.10–2.35
No. of reflections	13,126	28,377
*R*_*work*_^b^	0.1591	0.2270
*R*_*free*_^c^	0.1992	0.2784
No. of atoms		
Protein	4930	4893
Ligand	34	41
Water	/	55
B-factors		
Protein (Å^2^)	38.1	38.4
Ligand (Å^2^)	123.1	72.0
Water (Å^2^)	/	47.4
Rms deviations		
Bond lengths (Å)	0.012	0.013
Bond angles (°)	1.515	1.538
Ramachandran statistics		
Preferred region (%)	93.7	95.4
Allowed region (%)	6.3	4.3
Outliers (%)	0	0.3

a *R*_*sym*_ = Σ_*hkl*_Σ_*i*_│*I*_*i*_(*hkl*) − 〈*I*(*hkl*)〉│ / Σ_*hkl*_Σ_*i*_*I*_*i*_(*hkl*), where 〈*I*(*hkl*)〉 is the average intensity of equivalent reflections and the sum is extended over all measured observations for all unique reflections.

b *R*_*work*_ = Σ_*hkl*_(|* F*_o_ | − |* F*_c_ |) / Σ_*hkl*_ |* F*_o_ |, where |* F*_o_ | is the observed and |* F*_c_ | the calculated structure factor amplitude of a reflection.

c *R*_*free*_ was calculated by randomly omitting 5% of the observed reflections before refinement.

**Table 2:** Dissociation constants and thermodynamic parameters from ITC titrations. Results obtained with different aminoglycosides and APH(2″)-IVa are expressed as mean ± standard deviation from two (for kanamycin B and G418) or three (for all the other aminoglycosides) independent experiments. MW and N_CNO_ represent the molecular weight and number of non-hydrogen atoms of aminoglycosides, respectively. *K*_d_ values were calculated from ITC-derived *K*_a_. The value of − TΔS was determined at 25 °C. In the table, aminoglycosides are ranked from the highest to the lowest affinity for APH(2″)-IVa. Binding isotherms and fits are given in Fig. S1. Dissociation constants and thermodynamic parameters from ITC titrations. Results obtained with different aminoglycosides and APH(2″)-IVa are expressed as mean ± standard deviation from two (for kanamycin B and G418) or three (for all the other aminoglycosides) independent experiments. MW and N_CNO_ represent the molecular weight and number of non-hydrogen atoms of aminoglycosides, respectively. *K*_d_ values were calculated from ITC-derived *K*_a_. The value of − TΔS was determined at 25 °C. In the table, aminoglycosides are ranked from the highest to the lowest affinity for APH(2″)-IVa. Binding isotherms and fits are given in Fig. S1.

Aminoglycosides	MW (g mol^− 1^)	N_CNO_	*K*_d_ (μM)	Δ*G* (kcal mol^− 1^)	Δ*H* (kcal mol^− 1^)	− TΔ*S* (kcal mol^− 1^)
Sisomicin	447.5	31	2.3 ± 1.6	− 7.8 ± 0.4	− 1.8 ± 0.1	− 6.0 ± 0.4
Tobramycin	467.5	32	3.9 ± 0.6	− 7.4 ± 0.1	− 3.7 ± 0.6	− 3.7 ± 0.7
Kanamycin B	483.5	33	5.7 ± 1.1	− 7.2 ± 0.1	− 3.8 ± 0.2	− 3.4 ± 0.4
Gentamicin	463.6	33	9.7 ± 5.0	− 6.9 ± 0.3	− 2.5 ± 0.2	− 4.4 ± 0.3
G418	496.6	34	27.5 ± 3.3	− 6.2 ± 0.1	− 4.8 ± 0.1	− 3.0 ± 2.3
Paromomycin	615.6	42	63.6 ± 29.0	− 5.8 ± 0.3	− 2.5 ± 0.2	− 3.2 ± 0.4
Kanamycin A	484.5	33	79.9 ± 6.2	− 5.6 ± 0.1	− 5.5 ± 0.3	− 0.1 ± 0.2
Amikacin	585.6	40	82.2 ± 8.9	− 5.6 ± 0.1	− 4.0 ± 0.1	− 1.6 ± 0.1

**Table 3:** Steady state kinetic parameters and dissociation constants for aminoglycosides. Kinetic parameters were obtained at saturating concentration of MgATP (2.5 mM) and variable concentrations of aminoglycoside (0 to 100 μM) with APH(2″)-IVa. Results are expressed as mean ± standard deviation from at least two independent experiments. Aminoglycosides are ranked from the highest to the lowest catalytic efficiency (*k*_cat_/*K*_m_). For comparison, values obtained by Toth *et al.*[5] are mentioned in brackets, and the *K*_d_ values measured by ITC are shown.

Aminoglycosides	*k*_cat_ (s^− 1^)	*K*_m_ (μM)	*k*_cat_/*K*_m_ (μM^− 1^ s^− 1^)	*K*_d_ (μM)	*K*_d_/*K*_m_
Kanamycin A	0.90 ± 0.02	4.0 ± 0.5	0.225	79.9 ± 6.2	20.0
(0.92 ± 0.01)	(3.3 ± 0.3)	(0.279)	–	–
G418	0.49 ± 0.01	2.2 ± 0.4	0.223	27.5 ± 3.3	12.5
Sisomicin	0.73 ± 0.02	3.5 ± 0.6	0.209	2.3 ± 1.6	0.7
Gentamicin	0.47 ± 0.01	3.3 ± 0.6	0.142	9.7 ± 5.0	2.9
Kanamycin B	1.69 ± 0.11	12.5 ± 2.9	0.135	5.7 ± 1.1	0.5
Tobramycin	1.64 ± 0.08	13.2 ± 2.2	0.124	3.9 ± 0.6	0.3
(1.80 ± 0.03)	(1.5 ± 0.1)	(1.200)	–	–
Amikacin	na	na	na	82.2 ± 8.9	–
(0.15 ± 0.01)	(98.0 ± 15.0)	(0.002)	–	–
Paromomycin	na	na	na	63.6 ± 29.0	–

na: no detectable activity (< 0.001 s^− 1^), even by increasing the enzyme concentration up to 5 μM.

**Table 4:** Kinetic parameters determined from transient kinetic experiments. Transient amplitudes (*A*_burst_ and *A*_lag_), transient rate constants (*k*_burst_ and *k*_lag_) and steady state rate constants (*k*_ss_) determined from the *total* (quench–flow) or *free* (stopped–flow) ADP time courses are reported. Standard errors are from fitting of single curves composed of 26–29 (quench–flow) or 512 (stopped–flow) time points. Experimental conditions are given in Fig. 6.

Aminoglycosides	Quench–flow			Stopped–flow		
*A*_burst_ (mol/mol)	*k*_burst_ (s^− 1^)	*k*_ss_ (s^− 1^)	*A*_lag_ (mol/mol)	*k*_lag_ (s^− 1^)	*k*_ss_ (s^− 1^)
Kanamycin A	0.38 ± 0.04	3.4 ± 0.7	0.77 ± 0.02	− 0.35 ± 0.01	3.4 ± 0.1	0.70 ± 0.01
G418	0.34 ± 0.11	2.0 ± 1.1	0.35 ± 0.04	− 0.31 ± 0.01	1.6 ± 0.1	0.33 ± 0.01
Sisomicin	0.48 ± 0.07	2.0 ± 0.4	0.50 ± 0.02	− 0.44 ± 0.01	1.6 ± 0.1	0.48 ± 0.01
Tobramycin	0.44 ± 0.05	2.1 ± 0.4	1.08 ± 0.02	− 0.48 ± 0.01	3.3 ± 0.1	1.05 ± 0.01

## References

[1] Michael C.A., Dominey-Howes D., Labbate M. (2014). The antimicrobial resistance crisis: causes, consequences, and management. Front. Public Health.

[2] Ramirez M.S., Tolmasky M.E. (2010). Aminoglycoside modifying enzymes. Drug Resist. Updat..

[3] Toth M., Chow J.W., Mobashery S., Vakulenko S.B. (2009). Source of phosphate in the enzymic reaction as a point of distinction among aminoglycoside 2″-phosphotransferases. J. Biol. Chem..

[4] Tsai S.F., Zervos M.J., Clewell D.B., Donabedian S.M., Sahm D.F., Chow J.W. (1998). A new high-level gentamicin resistance gene, *aph*(*2*″)*-Id*, in *Enterococcus* spp.. Antimicrob. Agents Chemother..

[5] Toth M., Frase H., Antunes N.T., Smith C.A., Vakulenko S.B. (2010). Crystal structure and kinetic mechanism of aminoglycoside phosphotransferase-2″-IVa. Protein Sci..

[6] Shi K., Houston D.R., Berghuis A.M. (2011). Crystal structures of antibiotic-bound complexes of aminoglycoside 2″-phosphotransferase IVa highlight the diversity in substrate binding modes among aminoglycoside kinases. Biochemistry.

[7] Gutfreund H. (1995). Kinetics for the Life Sciences.

[8] Wu L., Serpersu E.H. (2009). Deciphering interactions of the aminoglycoside phosphotransferase(3′)-IIIa with its ligands. Biopolymers.

[9] Baker N.A., Sept D., Joseph S., Holst M.J., McCammon J.A. (2001). Electrostatics of nanosystems: application to microtubules and the ribosome. Proc. Natl. Acad. Sci. U. S. A..

[10] Dolinsky T.J., Czodrowski P., Li H., Nielsen J.E., Jensen J.H., Klebe G., Baker N.A. (2007). PDB2PQR: expanding and upgrading automated preparation of biomolecular structures for molecular simulations. Nucleic Acids Res..

[11] Phillips J.C., Braun R., Wang W., Gumbart J., Tajkhorshid E., Villa E., Chipot C., Skeel R.D., Kalé L., Schulten K. (2005). Scalable molecular dynamics with NAMD. J. Comput. Chem..

[12] Martyna G.J., Tobias D.J., Klein M.L. (1994). Constant pressure molecular dynamics algorithms. J. Chem. Phys..

[13] Feller S.E., Zhang Y., Pastor R.W., Brooks B.R. (1995). Constant pressure molecular dynamics simulation: the Langevin piston method. J. Chem. Phys..

[14] MacKerell A.D., Banavali N., Foloppe N. (2000). Development and current status of the CHARMM force field for nucleic acids. Biopolymers.

[15] Humphrey W., Dalke A., Schulten K. (1996). VMD: visual molecular dynamics. J. Mol. Graph..

[16] Essmann U., Perera L., Berkowitz M.L., Darden T., Lee H., Pedersen L.G. (1995). A smooth particle mesh Ewald method. J. Chem. Phys..

[17] Hénin J., Chipot C. (2004). Overcoming free energy barriers using unconstrained molecular dynamics simulations. J. Chem. Phys..

[18] Toth M., Vakulenko S., Smith C.A. (2010). Purification, crystallization and preliminary X-ray analysis of *Enterococcus casseliflavus* aminoglycoside-2″-phosphotransferase-IVa. Acta Crystallogr. Sect. F Struct. Biol. Cryst. Commun..

[19] Gelin M., Delfosse V., Allemand F., Hoh F., Sallaz-Damaz Y., Pirocchi M., Bourguet W., Ferrer J.L., Labesse G., Guichou J.F. (2015). Combining “dry” co-crystallization and in situ diffraction to facilitate ligand screening by X-ray crystallography. Acta Crystallogr. D Biol. Crystallogr..

[20] Kabsch W. (2010). XDS. Acta Crystallogr. D Biol. Crystallogr..

[21] Collaborative Computational Project (1994). Number 4, the CCP4 suite: programs for protein crystallography. Acta Crystallogr. D Biol. Crystallogr..

[22] Emsley P., Lohkamp B., Scott W.G., Cowtan K. (2010). Features and development of Coot. Acta Crystallogr. D Biol. Crystallogr..

[23] Murshudov G.N., Vagin A.A., Dodson E.J. (1997). Refinement of macromolecular structures by the maximum-likelihood method. Acta Crystallogr. D Biol. Crystallogr..

[24] Painter J., Merritt E.A. (2006). Optimal description of a protein structure in terms of multiple groups undergoing TLS motion. Acta Crystallogr. D Biol. Crystallogr..

[25] Schüttelkopf A.W., van Aalten D.M.F. (2004). PRODRG: a tool for high-throughput crystallography of protein–ligand complexes. Acta Crystallogr. D Biol. Crystallogr..

[26] Adams P.D., Afonine P.V., Bunkóczi G., Chen V.B., Davis I.W., Echols N., Headd J.J., Hung L.-W., Kapral G.J., Grosse-Kunstleve R.W., McCoy A.J., Moriarty N.W., Oeffner R., Read R.J., Richardson D.C., Richardson J.S., Terwilliger T.C., Zwart P.H. (2010). PHENIX: a comprehensive Python-based system for macromolecular structure solution. Acta Crystallogr. D Biol. Crystallogr..

[27] Lallemand P., Leban N., Kunzelmann S., Chaloin L., Serpersu E.H., Webb M.R., Barman T., Lionne C. (2012). Transient kinetics of aminoglycoside phosphotransferase(3′)-IIIa reveals a potential drug target in the antibiotic resistance mechanism. FEBS Lett..

[28] Kunzelmann S., Webb M.R. (2009). A biosensor for fluorescent determination of ADP with high time resolution. J. Biol. Chem..

[29] Boehr D.D., Farley A.R., Wright G.D., Cox J.R. (2002). Analysis of the pi–pi stacking interactions between the aminoglycoside antibiotic kinase APH(3′)-IIIa and its nucleotide ligands. Chem. Biol..

[30] Özen C., Serpersu E.H. (2004). Thermodynamics of aminoglycoside binding to aminoglycoside-3′-phosphotransferase IIIa studied by isothermal titration calorimetry. Biochemistry.

[31] Özen C., Malek J.M., Serpersu E.H. (2006). Dissection of aminoglycoside–enzyme interactions: a calorimetric and NMR study of neomycin B binding to the aminoglycoside phosphotransferase(3′)-IIIa. J. Am. Chem. Soc..

[32] Özen C., Norris A.L., Land M.L., Tjioe E., Serpersu E.H. (2008). Detection of specific solvent rearrangement regions of an enzyme: NMR and ITC studies with aminoglycoside phosphotransferase(3′)-IIIa. Biochemistry.

[33] Hegde S.S., Dam T.K., Brewer C.F., Blanchard J.S. (2002). Thermodynamics of aminoglycoside and acyl-coenzyme a binding to the *Salmonella enterica* AAC(6′)-Iy aminoglycoside N-acetyltransferase. Biochemistry.

[34] Wright E., Serpersu E.H. (2005). Enzyme–substrate interactions with an antibiotic resistance enzyme: aminoglycoside nucleotidyltransferase(2″)-Ia characterized by kinetic and thermodynamic methods. Biochemistry.

[35] Wright E., Serpersu E.H. (2006). Molecular determinants of affinity for aminoglycoside binding to the aminoglycoside nucleotidyltransferase(2″)-Ia. Biochemistry.

[36] Norris A.L., Özen C., Serpersu E.H. (2010). Thermodynamics and kinetics of association of antibiotics with the aminoglycoside acetyltransferase (3)-IIIb, a resistance-causing enzyme. Biochemistry.

[37] Norris A.L., Serpersu E.H. (2010). Interactions of coenzyme A with the aminoglycoside acetyltransferase (3)-IIIb and thermodynamics of a ternary system. Biochemistry.

[38] Norris A.L., Serpersu E.H. (2011). Antibiotic selection by the promiscuous aminoglycoside acetyltransferase-(3)-IIIb is thermodynamically achieved through the control of solvent rearrangement. Biochemistry.

[39] Jing X., Wright E., Bible A.N., Peterson C.B., Alexandre G., Bruce B.D., Serpersu E.H. (2012). Thermodynamic characterization of a thermostable antibiotic resistance enzyme, the aminoglycoside nucleotidyltransferase (4′). Biochemistry.

[40] Hopkins A.L., Groom C.R., Alex A. (2004). Ligand efficiency: a useful metric for lead selection. Drug Discov. Today.

[41] Schultes S., de Graaf C., Haaksma E.E.J., de Esch I.J.P., Leurs R., Krämer O. (2010). Ligand efficiency as a guide in fragment hit selection and optimization. Drug Discov. Today Technol..

[42] Romanowska J., Reuter N., Trylska J. (2013). Comparing aminoglycoside binding sites in bacterial ribosomal RNA and aminoglycoside modifying enzymes. Proteins.

[43] Shakya T., Stogios P.J., Waglechner N., Evdokimova E., Ejim L., Blanchard J.E., McArthur A.G., Savchenko A., Wright G.D. (2011). A small molecule discrimination map of the antibiotic resistance kinome. Chem. Biol..

[44] Segel I.H., Segel I.H. (1975). Rapid equilibrium bireactant and terreactant systems. Enzyme Kinetics: Behaviour and Analysis of Rapid Equilibrium and Steady-state Enzyme Systems.

[45] Lallemand P., Chaloin L., Roy B., Barman T., Bowler M.W., Lionne C. (2011). Interaction of human 3-phosphoglycerate kinase with its two substrates: is substrate antagonism a kinetic advantage?. J. Mol. Biol..

[46] Kim C., Haddad J., Vakulenko S.B., Meroueh S.O., Wu Y., Yan H., Mobashery S. (2004). Fluorinated aminoglycosides and their mechanistic implication for aminoglycoside 3′-phosphotransferases from Gram-negative bacteria. Biochemistry.

[47] Kim C., Cha J.Y., Yan H., Vakulenko S.B., Mobashery S. (2006). Hydrolysis of ATP by aminoglycoside 3′-phosphotransferases: an unexpected cost to bacteria for harboring an antibiotic resistance enzyme. J. Biol. Chem..

[48] Shi K., Berghuis A.M. (2012). Structural basis for dual nucleotide selectivity of aminoglycoside 2″-phosphotransferase IVa provides insight on determinants of nucleotide specificity of aminoglycoside kinases. J. Biol. Chem..

