# Supplementary material for: Aminoglycoside binding and catalysis specificity of aminoglycoside 2″-phosphotransferase IVa: A thermodynamic, structural and kinetic study
Source: Biochim Biophys Acta. 2016 Apr;1860(4):802–13. doi: 10.1016/j.bbagen.2016.01.016 (PMC4769084; doi:10.1016/j.bbagen.2016.01.016)
Supplement: Fig. S1 — Binding isotherms and fits obtained with (a) kanamycin A, (b) kanamycin B, (c) sisomicin, (d) tobramycin, (e) paromomycin, (f) amikacin, (g) gentamicin and (h) G418. (i) Thermodynamic parameters obtained: ∆ G (gray), ∆ H (dashed) and − T ∆ S (white). Data are shown as mean ± standard deviation from 2–3 independent repetitions. From left to right, aminoglycosides are ranked from the highest to the lowest absolute value of ∆ G. [file mmc1.docx]

**Fig. S1.** Binding isotherms and fits obtained with (a) kanamycin A, (b) kanamycin B, (c) sisomicin, (d) tobramycin, (e) paromomycin, (f) amikacin, (g) gentamicin and (h) G418. (i) Thermodynamic parameters obtained: Δ*G* (grey), Δ*H* (dashed) and –TΔ*S* (white). Data are shown as mean ± standard deviation from 2-3 independent repetitions. From left to right, aminoglycosides are ranked from the highest to the lowest absolute value of Δ*G*.
