# Supplementary material for: Aminoglycoside binding and catalysis specificity of aminoglycoside 2″-phosphotransferase IVa: A thermodynamic, structural and kinetic study
Source: Biochim Biophys Acta. 2016 Apr;1860(4):802–13. doi: 10.1016/j.bbagen.2016.01.016 (PMC4769084; doi:10.1016/j.bbagen.2016.01.016)

**Fig. S2.** Structural analysis of hypothetical sisomicin binding site. Panels a and d show the 2*F*_o_ – *F*_c_ omit maps contoured at 1 σ (gray) obtained by X-ray crystallography for chain A and chain B, respectively. The density in chain A (a) only permits to position the ring A of sisomicin. Stabilizing interactions are detailed (b, c). The density in chain B (d) shows a different packing-induced binding position of sisomicin (e, f) which forms a stabilizing interaction with the backbone of Ser136 belonging to a symmetric protein of the unit cell. (g) Predicted binding mode (docking pose) of sisomicin in the 4DFB protein after removal of kanamycin A. Main interactions are figured (h) and detailed (i).


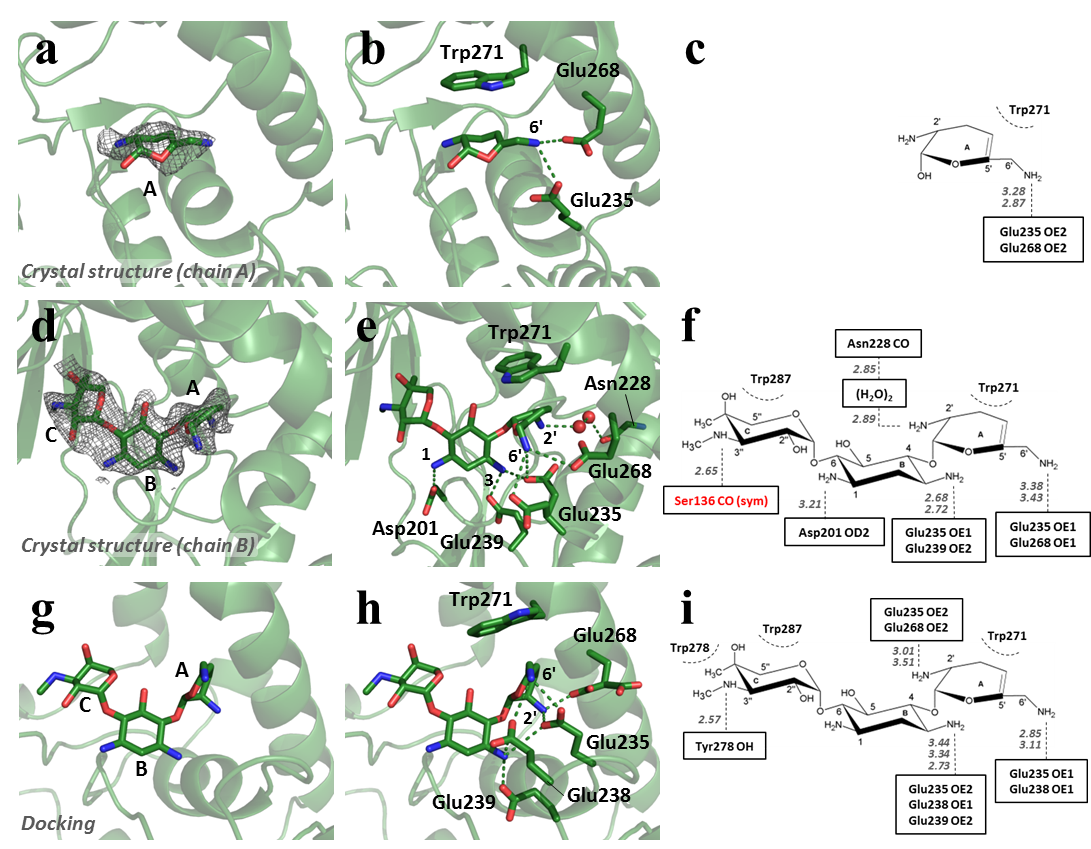

Supplement: Fig. S2 — Structural analysis of hypothetical sisomicin binding site. Panels a and d show the 2Fo − Fc omit maps contoured at 1 σ (gray) obtained by X-ray crystallography for chain A and chain B, respectively. The density in chain A (a) only permits the positioning of the ring A of sisomicin. Stabilizing interactions are detailed (b, c). The density in chain B (d) shows a different packing-induced binding position of sisomicin (e, f) which forms a stabilizing interaction with the backbone of Ser136 belonging to a symmetric protein of the unit cell. (g) Predicted binding mode (docking pose) of sisomicin in the 4DFB protein after removal of kanamycin A. Main interactions are figured (h) and detailed (i). [file mmc2.docx]
